# The Evolving Diagnostic and Genetic Landscapes of Autism Spectrum Disorder

**DOI:** 10.3389/fgene.2016.00065

**Published:** 2016-04-26

**Authors:** Mark N. Ziats, Owen M. Rennert

**Affiliations:** ^1^Laboratory of Clinical and Developmental Genomics, National Institute of Child Health and Human Development, National Institutes of Health, Bethesda, MD, USA; ^2^Department of Physiology, Development and Neuroscience, University of CambridgeCambridgeshire, UK; ^3^Medical Scientist Training Program, Baylor College of MedicineHouston, TX, USA

**Keywords:** autistic disorder, autistic spectrum disorder, genetics, medical, pathways, phenotype

## Abstract

The autism spectrum disorders (ASD) are a heterogeneous set of neurodevelopmental syndromes defined by impairments in verbal and non-verbal communication, restricted social interaction, and the presence of stereotyped patterns of behavior. The prevalence of ASD is rising, and the diagnostic criteria and clinical perspectives on the disorder continue to evolve in parallel. Although the majority of individuals with ASD will not have an identifiable genetic cause, almost 25% of cases have identifiable causative DNA variants. The rapidly improving ability to identify genetic mutations because of advances in next generation sequencing, coupled with previous epidemiological studies demonstrating high heritability of ASD, have led to many recent attempts to identify causative genetic mutations underlying the ASD phenotype. However, although hundreds of mutations have been identified to date, they are either rare variants affecting only a handful of ASD patients, or are common variants in the general population conferring only a small risk for ASD. Furthermore, the genes implicated thus far are heterogeneous in their structure and function, hampering attempts to understand shared molecular mechanisms among all ASD patients; an understanding that is crucial for the development of targeted diagnostics and therapies. However, new work is beginning to suggest that the heterogeneous set of genes implicated in ASD may ultimately converge on a few common pathways. In this review, we discuss the parallel evolution of our diagnostic and genetic understanding of autism spectrum disorders, and highlight recent attempts to infer common biology underlying this complicated syndrome.

## Clinical phenotype and incidence

Autism was first described 70 years ago by the American child psychiatrist Kanner (1943). While originally reported by Kanner as an isolated syndrome with the core components being “obsessive insistence on the preservation of sameness” and “autistic aloneness,” autism was considered mainly as a childhood form of schizophrenia for more than 30 years (Eisenberg and Kanner, 1956). Autism was first formally recognized as its own clinical diagnostic entity in 1980, defined as encompassing three essential features: impairment in communication, lack of interest in other people, and “bizarre” behaviors (American Psychiatric Association, 1980). Since that time, the criteria required to obtain a diagnosis of ASD, and its relation to other similar disorders such as Asperger's and Rett syndrome, have changed multiple times—reflecting both the clinical heterogeneity of the disorder and the poor understanding of its underlying pathophysiology.

The most recent definition of ASD recognizes abnormalities in two clinical domains: ‘social and communication defects’ and “fixed interests and repetitive behaviors” (American Psychiatric Association, 2013). All of the following three symptoms describing persistent deficits in social interaction and communication must be present for a diagnosis of ASD to be made: (i) problems reciprocating social or emotional interaction, inability to initiate an interaction, and problems with shared attention or sharing of emotions and interests with others; (ii) problems maintaining relationships and problems adjusting to different social expectations; and (iii) nonverbal communication problems such as abnormal eye contact, posture, facial expressions, tone of voice, and gestures, as well as an inability to understand these. Additionally, these interaction/communication deficits cannot be better accounted for by general developmental delay. Two of the four following symptoms related to restricted and repetitive behavior must also be present: (i) stereotyped or repetitive speech, motor movements, or use of objects; (ii) excessive adherence to routines, ritualized patters of verbal or nonverbal behavior, or excessive resistance to change; (iii) highly restricted interests that are abnormal in intensity or focus; and (iv) hyper- or hypo-reactivity to sensory input or unusual interest in sensory aspects of the environment.

Furthermore, the severity of each symptom must be defined based on the level of support required for that symptom, in an attempt to more thoroughly capture the “spectrum” nature of the disease. In all cases, symptoms must have been present in early childhood (even if initially unrecognized) although they may not become fully manifest until later in life when social demands exceed capacities. The symptoms must impair everyday functioning, and cannot be better described by another diagnosis.

Autism spectrum disorders are one of the most common neurodevelopmental problems affecting children in the Western world. The most recent estimates have shown that ASD affects between 1 in 68 children (Centers for Disease Control Prevention, 2012) and perhaps as many as 1 in 50, (Blumberg et al., 2013) depending on the methodology employed. This represents a staggering 1.17–2% of all children. Boys are at least four times more likely to receive a diagnosis of ASD as compared to girls, (Centers for Disease Control Prevention, 2012) and this ratio increases significantly when only mildly affected children are considered (Gillberg et al., 2006). Furthermore, prevalence estimates have been increasing substantially in recent years—from 1 in 150 children in the year 2000—although it is unclear to what extent this represents a true biological increase or is a result of expanding diagnostic criteria and better clinical recognition of the disorder (Fombonne, 2009).

The costs associated with autism are similarly great. Economically, direct and indirect medical costs are estimated to be over $3.2 million dollars per person over his or her lifetime, or more than $34 billion dollars per year for all people with ASD (Moldin and Rubenstein, 2006). Perhaps more importantly, the emotional toll placed on parents and caregivers of children with autism is immense, unrelenting, and has a serious impact on family relationships, (Rao and Beidel, 2009) marriages, (Benson and Kersh, 2011), and a couples' future reproductive decisions (Selkirk et al., 2009).

Consequently, it is of upmost urgency to patients with ASD, their caregivers, and society at large that the underlying cause(s) of the disorder is/are understood. Doing so will enable the development of better, more specific diagnostic tests that can recognize ASD earlier in life, which has been shown to be important to improve long-term outcomes, (Howlin et al., 2009) can provide parents with an explanation for their child's symptoms, and may eventually enable the development of targeted therapeutics. Moreover, by understanding the mechanisms that lead to the neurobehavioral autistic phenotype, the field of human neuroscience as a whole can be advanced, as it will provide insights into the genetic and molecular basis of higher cognitive functioning.

However, the underlying pathophysiology of autism spectrum disorders have long been a mystery. Various hypothesis ranging from psychosocial to environmental have been purported, yet it was not until twin and sibling epidemiological studies were undertaken in the 1980s that the strong heritability of ASD began to be realized. Subsequently, a large amount of work has firmly established a significant genetic component to ASD's etiology.

## Genetic etiology

Evidence for a strong heritable risk of ASD was initially described in twin and sibling epidemiological studies of autism, (Folstein and Rutter, 1977) and has since been firmly established through multiple genetic approaches (Geschwind, 2011). It was first recognized that the risk of having a second child with autism was higher in families that already had one child with ASD than was the risk of having a child with ASD in the general population. Originally this recurrence risk was estimated to be 5% (compared with ~1% in the general population), although more recent estimates suggest it may be as high as 20% (Ozonoff et al., 2011). Following these initial observations, the first twin studies in ASD demonstrated a concordance rate approaching 90% in monozygotic twins and 10% in dizygotic twins (Ritvo et al., 1989; Steffenburg et al., 1989; Bailey et al., 1995; Smalley et al., 1998). Subsequently, larger studies have shown the dizygotic concordance rate to be at least greater than 20% (Hallmayer et al., 2011).

These observations, coupled with the identification of causative genetic mutations in monogenic disorders with autism as a component, such as Fragile X and Rett syndromes, (Pieretti et al., 1991; Amir et al., 1999) led to an ongoing effort to identify genetic causes of “idiopathic” ASD using a number of genomic approaches. As the technology behind these approaches has improved, the ability to identify mutations with incredible sensitivity and genomic resolution has resulted in over 700 genetic loci implicated in ASD to date (Freitag, 2007; Basu et al., 2009; Anney et al., 2010). However, as more genes and loci are identified, it is becoming increasingly clear that the genomic architecture of ASD is incredibly heterogeneous and complex, necessitating a functional integration in order to decipher common molecular mechanisms underlying ASD.

## Genomic architecture of ASD

The identification of genomic loci and individual genes disrupted in patients with ASD has progressed in tandem with the rapid development of sensitive genomic tools. Initially, microscopically-visible chromosomal aberrations were observed in patients with ASD who received karyotyping analysis. These case reports were variable, but a number of loci were repeatedly implicated, (Vorstman et al., 2006) including 7q11, 15q11–13, and 22q11.2—regions already associated with syndromes that had autistic symptoms as a component, and known to contain a number of critical neurodevelopmental genes and some of the first identified functional non-coding RNAs (Szafranski et al., 2010; Mabb et al., 2011).

Subsequently, the development of microarray technology such as comparative genomic hybridization (CGH), allowed the unbiased assessment of copy-number variation (CNV) across the whole genome at a resolution of as low as 100 kilobases (Alkan et al., 2011). The first of these analysis indicated that individuals with ASD had 10–20 times the number of CNVs as controls (Jacquemont et al., 2006; Sebat et al., 2007). Numerous studies have since used CGH or similar approaches to follow up and improve upon these initial reports with larger and more homogenous patient populations, with thousands of individuals with ASD having been analyzed to date.(Autism Genome Project Consortium et al., 2007; Christian et al., 2008; Marshall et al., 2008; Glessner et al., 2009; Itsara et al., 2010; Pinto et al., 2010; Cooper et al., 2011; Gilman et al., 2011; Sanders et al., 2011) These studies have consistently demonstrated that individuals with ASD have more CNVs than non-related controls. Furthermore, studies employing a family cohort model have been able to compare individuals with ASD to their parents and unaffected siblings, which has revealed that *de novo* CNVs in particular are more frequent in children with ASD.

Functionally, it was also shown that larger CNVs (i.e., affecting more genes) are associated with decreased cognition, (Girirajan et al., 2012) and that females with ASD tend to have larger CNVs than males with ASD, (Itsara et al., 2010; Sanders et al., 2011) suggesting they are somehow more “genetically tolerant” of these disruptions. Moreover, some of the identified loci result in nearly opposite phenotypes depending on whether they are duplicated or deleted (Jacquemont et al., 2011). Taken together, these functional CNV findings suggest that identification of the genes in these regions is not sufficient to understand the mechanisms underlying autism, as it appears that a finely-regulated dosage of each gene is necessary to avoid neurodevelopmental disorders such as ASD.

Despite the progress made with microarrays, the findings from these studies only identified CNVs in 5–15% of individuals with ASD, suggesting that other types of mutations must be operant in ASD as well. However, investigations at higher genomic resolution were traditionally limited to specific candidate genes until the recent widespread availability of next-generation sequencing (NGS) technologies and high resolution genome-wide single nucleotide polymorphism (SNP) microarrays. Since then, many large exome sequencing studies have been completed in ASD, encompassing more than 1000 affected individuals (O'Roak et al., 2011, 2012a,b; Klei et al., 2012; Kong et al., 2012; Neale et al., 2012; Sanders et al., 2012). In addition to identifying a number of high-confidence ASD candidate genes (likely representing 5–10% of ASD cases), these studies provided two other more broad insights into the functional genomics of ASD that are particularly noteworthy. First, with the exception of a few identified genes, there was very little replication of ASD candidates among the studies. This had led to the notion that many variants causative for the ASD phenotype are likely to be very rare or “private” mutations, which are unlikely to be found in more than one individual at the current scale with which these studies are conducted—suggesting the number of rare mutations that can impart substantial risk for ASD is much larger than originally suspected. Secondly, a meta-analysis of these studies at the group level showed that the average rate of mutations in individuals with ASD was not significantly different than control—or even unaffected siblings—unless the analysis was restricted to genes that are known to be expressed during human brain development (Sanders et al., 2012). This highlights the tissue- and human-specific nature of gene function, which underscores the importance of understanding the function of ASD candidate genes in the context of human brain development specifically, which remains a major challenge to the field.

Similarly, genome-wide association studies (GWAS) using high resolution SNP arrays, which in contrast are designed to assess for more common variants likely to increase autism risk less substantially (i.e., variants founds in greater than 1% of the population), have also not revealed a small set of genes likely to be commonly found in patients with ASD (Autism Genome Project Consortium et al., 2007; Wang et al., 2009; Weiss et al., 2009). While GWAS approaches have indeed shown the importance of common variants to ASD risk, (Gaugler et al., 2014) it has become increasingly clear that the potential number of genes likely able to confer moderately-sized risk for ASD is large and varied. In fact, statistical modeling based on published results of both rare and common variation have predicted that up to 1000–1500 genes may ultimately be found to be associated with ASD (Iossifov et al., 2012; Sanders et al., 2012). Therefore, understanding how such a large and varied number of genes can all be associated with one common clinical phenotype will end up being *the* major challenge to the field, once all implicated genes are identified.

Lastly, there is a growing appreciation that the presence of multiple mutations and/or inherited protective or risk alleles—each at different loci within one individual—may interact with each other to result in the emergent ASD phenotype, and that this may help explain the complex and heterogeneous nature of ASD genomics. For instance, a number of studies have described individuals with ASD who have more than one deleterious mutation, (Girirajan et al., 2010, 2012; Leblond et al., 2012) and the presence of more than one mutation correlates with an increased risk of developmental delay (Girirajan et al., 2012). Other studies have suggested certain inherited variants may be protective against other ASD-causing mutations, especially in females (Robinson et al., 2013). While the identification of multiple mutations within individuals is becoming a relatively straightforward task, the challenge of understanding how combinations of susceptibility genes interact during human brain development to cause disease (epistasis) has only begun to be explored.

## From ASD candidate genes to candidate pathways

Finally, several pathway analyses have been performed using either genetic or transcriptome data to gain insight into the biological functions associated with ASD candidate genes. For instance, O'Roak et al. analyzed protein-interaction networks among genes implicated in ASD via whole-exome sequencing studies, and identified that *de novo* mutations in ASD patients are overrepresented among proteins involved in a chromatin remodeling network (O'Roak et al., 2012b). Similarly, Gilman et al. demonstrated that CNVs identified in autistic patients are enriched for genes involved in a molecular network related to synaptogenesis, axon guidance, and neuronal motility (Gilman et al., 2011).

A number of more recent studies have attempted to integrate autism candidate genes with known human brain gene expression patterns. Ben-David and Shifman attempted to assess for differences between rare and common ASD candidate genes by studying their co-expression relationships in adult human brain. They discovered these genes were both related to modules involved with synaptogenesis and neuronal plasticity, and that are expressed in areas associated with learning, memory, and sensory perception (Ben-David and Shifman, 2012). The same authors also recently analyzed the neurodevelopmental expression of ASD candidate genes that had been discovered in cohorts as *de novo* mutations, and demonstrated that these genes appear to relate to networks involved in transcription regulation and chromatin remodeling processes (Ben-David and Shifman, 2013). Using the same neurodevelopmental transcriptional profiling dataset, two other studies recently attempted to infer convergent molecular pathways and neural circuits among various sets of ASD candidate genes. An assessment of the co-expression relationships among brain gene expression seeded with “high confidence” ASD candidate genes suggested they are most highly co-expressed during the mid-fetal developmental period, and in layer 5/6 cortical projection neurons (Willsey et al., 2013). A similar study assessing a broader list of ASD candidates suggested that the heterogeneous genes may ultimately converge upon the molecular pathways of transcriptional regulation in early development, and synaptogenesis in later childhood (Parikshak et al., 2013). While such studies are mainly exploratory in nature, the provide some of the first insight that a few common mechanisms may ultimately relate the heterogeneous set of ASD candidate genes to one another.

## Conclusion

In summary, a diagnosis of autism spectrum disorder is common, and has an incredibly profound impact on individuals with ASD, their families, and society at large. Because the underlying cause(s) of ASD are not understood, specific diagnostic tests and therapeutic strategies are unavailable. The evolution of ASD's clinical definition is indicative of the heterogeneous and complex nature of the disorder. While ASD has been shown to have a significant genetic etiological component, recent attempts to discover genes associated with ASD risk have implicated hundreds (now approaching thousands) of heterogeneous loci. Moreover, this estimate does not even consider the emerging role of non-coding RNA variants (Barry, 2014). Consequently, attempts to understand how this diverse set of genes relates to the underlying molecular mechanisms and subsequent cellular neuropathology of ASD remains poorly understood.

This body of evidence suggests that while identification of candidate genes in ASD is a critical first step toward understanding the genetic etiology of this disorder, a comprehensive, disorder-specific understanding of the molecular mechanisms cannot be realized until the functional genomics of ASD candidate genes are properly understood in the context of human brain development. Therefore, studies that attempt to reconcile the heterogeneous and varied nature of ASD genomics are necessary to move the field forward toward a common understanding of the mechanisms underlying the development of ASD.

## Author contributions

OR and MZ conceived and designed the review. MZ wrote the review. OR and MZ read and edited the review.

### Conflict of interest statement

The authors declare that the research was conducted in the absence of any commercial or financial relationships that could be construed as a potential conflict of interest.
